# Analyzing Older Adults’ Perceived Values of Using Smart Bracelets by Means–End Chain

**DOI:** 10.3390/healthcare8040494

**Published:** 2020-11-18

**Authors:** Mei-Yuan Jeng, Tsu-Ming Yeh, Fan-Yun Pai

**Affiliations:** 1 Department of Leisure Recreation and Management, Da-Yeh University, Changhua 515, Taiwan; mei521113@yahoo.com.tw; 2 Department of Industrial Engineering and Management, National Quemoy University, Kinmen 892, Taiwan; 3 Department of Business Administration, National Changhua University of Education, Changhua 500, Taiwan

**Keywords:** older adults, smart healthcare wearable devices, means–end chain, value, usage intention

## Abstract

To cope with the demands for medical care in an aging society, smart healthcare wearable devices that can measure physiological signals are being regarded as the primary tools in medical care programs, allowing the users to acquire basic health data. Although the smart healthcare wearable devices could be applied to disease management and prevention that could help older adults control their health, older adults must be willing and able to use and continue to use them. In this research, interviews conducted through means–end chain (MEC) and laddering were used to guide the older adults step-by-step by explaining abstract ideas and emphasizing value in their perceptions of specific attributes. A “hierarchical value map” was further constructed to confirm the perceived value of smart healthcare wearable devices to older adults. The research results showed that, in terms of attribute functions, seniors believed that smart bracelets in mobile health devices should have the attributes of safe use, real-time information feedback, correct data, comfortable wear, and clear screen. In terms of consequent benefits, older adults can use smart bracelets in mobile health devices to gain benefits in learning about smart products, understanding technology applications, increasing health awareness and relaxation, and satisfying curiosity. In terms of value goals, older adults want to achieve the value goals of a sense of social belonging, improved quality of life, and healthier bodies. Health is the most important thing for older adults, but previous research has often focused on the use of equipment for physical examinations; relatively few studies have allowed older adults to experience the equipment personally. The device can provide the ultimate value of long-term health promotion for older adults.

## 1. Introduction

As a result of advances in medical technology, an emphasis on health care, and improvements in the living environment, the average human lifespan is now significantly longer in both developing and developed countries [1,2]. Accordingly, issues related to the social phenomenon of aging have become important to countries around the world [3]. In 2019, 1 in 11 people in the world were aged 65 or older, and 1 in 6 will be aged 65 or older by 2050, accounting for more than 16% of the total population [4,5]. In 2002, the United Nations adopted the Madrid International Plan of Action on Ageing, which called for countries, regardless of region, to prioritize aging in their policy actions [1]. In addition to the provision of social welfare, such as health care and medical care, how to enable older adults to live independently and with self-respect so as not to become a burden on society, and even provide them with wisdom and other contributions, has become an important source of national competitiveness [6].

Aging populations are often associated with an increased risk of disease and deterioration of physiological functions [7], including reduced functioning of the brain and cardiovascular, skeletal, and muscular systems [8]. Although physiological deterioration is associated with the aging process itself, a lack of physical activity has been identified as a risk factor for cardiovascular disease and other chronic diseases that are common among older adults [9]. Hallal et al. [10] found that approximately 31% of adults worldwide are physically inactive. Inactivity is currently ranked as the fourth leading cause of death in the world, and there are significant declines in activity levels after retirement [11]. If the decline of function in older adults can be delayed through early detection and preventive measures to reduce risk [7], so that older adults are able to monitor their physical condition at all times and stay as active and independent as possible, the prevention or reduction of health problems can be considerably improved [12].

Following the rapid development and diversification of technology products, new human–machine technology provides different ways to communicate with, provide entertainment to, care for, and interact with older adults [13]. Mobile health devices (e.g., smartphones, bracelets, and watches) make use of mobile technology, wireless devices, and sensors [14]. When wearing these devices during normal daily activities, people can derive accurate information about their physiological, psychological, emotional, and environmental states by outputting data from the sensor and additional self-reported data [15]. Users’ health status, including psychophysiological data such as sleep, calories burned, heart rate, brain activity, and muscle activity, can be tracked by monitoring individual behavior [16]. Such devices for health-related monitoring have greater precision than traditional methods; they are more suitable for long-term use [7].

Since Apple launched the Apple Watch, the media has started to focus on mobile health devices. Facing the problems of declining birthrates and an aging society, mobile health devices play the roles of caring for and monitoring older adults [17]. However, existing research has not explored how older adults can use mobile health devices to promote and maintain healthy lifestyles. Therefore, the present study uses the means–end chain (MEC) approach to explore the value of mobile health devices to older adults, identifying the attributes, consequences, and values of mobile health devices and establishing the relationship between them. This study seeks to understand changes in the value orientations and behavioral decision making among older adults as a basis for relevant units to meet the needs of older adults and encourage them to live an active life and as a basis for further developing these technologies and services according to the needs and preferences of older adults.

## 2. Literature Review

### 2.1. Demand for Mobile Health Devices in an Aging Society

The impact of aging on society is visible not only in a decline in the overall productive workforce, but also in the serious effects of illnesses in older adults on the finances and lives of family members [6]. The past model of old-age care that relied on family members or caregivers to detect symptoms passively could be replaced with wearable mobile health devices for the daily health monitoring in older adults. This combination of mobile technology communication with data uploading, using the huge amount of data generated by continuous monitoring, could significantly improve the health of older adults and would be the key to geriatric preventive medicine [7]. In the past ten years, older adults have become the fastest-growing group of people using information technology products [18]. A nationwide survey in the United States showed that the rate of use of digital health technology by older adults over 65 years of age increased from 21% in 2011 to 25% in 2014 [19]. Sixty-two percent of chronic patients use the monitoring provided by health electronic technology to track their health status. Although some scholars have argued that the use of information technology will weaken the face-to-face social interaction between older adults [20], other scholars have argued that information technology can promote social participation and that social participation is an important factor affecting the health of elderly people [21].

The wearable mobile health devices that are generally familiar to older adults are simple and inexpensive smart bracelets. Wearing these devices, older adults can monitor their physiological status during activity, enabling them to maintain a healthy routine. These devices also feature heart rate sensors and positioning systems and provide communication by wired or wireless transmission, providing a convenient channel for two-way interaction with health care personnel. This increases the freedom of care recipients in their activities and self-management of diseases, greatly benefiting their health [15]. For older adults, the pedometer and sleep monitoring functions are the main physiological functions of their smart bracelets. In terms of mental state and quality of life, maintaining a happy mood and recording everyday life occurrences are also the functions of mobile health devices frequently used by older adults [7].

### 2.2. Mobile Health Devices

A mobile health device (mHealth) is a small communication device that works with a smartphone or a tablet computer to help people manage their health at any time, reducing the use of medical resources and improving quality of life. Medical smart wearable devices have emerged as products with commercial potential in recent years due to the increased demand for chronic disease monitoring, long-term care, and self-directed health management [15,22]. Wang et al. [14] pointed out that smart wearable devices have the function of sensor connectivity, with the capability to sense, collect, display, or transmit information over time, and the ability to transmit data over the Internet and can be used for long periods of time without interfering with daily routines. The potential for the development of new services based on such technology is tremendous. In 2014, over 75% of people aged 65 or older in the United States had a mobile phone, and over 50% used smartphones or tablets [23], while in the United Kingdom in 2012, roughly 50% used the Internet, which is projected to rise to 90% by 2020 [24]. The number of people worldwide owning a smartphone reached 2.1 billion in 2016, and the numbers are expected to rise to 2.5 billion by 2020 [25]. The number of downloads of health and fitness apps reached 165 million in 2015 and is increasing rapidly [26], demonstrating the feasibility of mobile health devices as personal health monitors and encouraging self-health management behaviors.

The application of mobile health in disease management has been demonstrated in clinical studies on diabetes control [27], depression treatment [28], and hypertension control [29]. Behavioral change applications include diet control using a personal digital assistant (PDA) application to reduce calorie intake [30]. Further, the use of smartphone apps and workout platforms for diet improvement, physical activity, and the prevention of sedentary behavior has been shown to be effective [31]. Tablet applications also offer balance and strength training, while smartphones offer a social element to physical activity [32].

Smart wearable products include smart glasses, smart bracelets, smart watches, smart apparel, smart rings, smart shoes and socks, and smart belts. Their functions include sleep monitoring, heart rate measurement, calorie consumption records, pedometers, GPS location, and odometers. In the current mobile health devices market, because smart watches and smart bracelets are easier to obtain, public awareness and acceptance of these devices are also higher. Therefore, the present study focuses on smart bracelets to explore the experiences of older adults wearing smart wearable products.

### 2.3. Value

Value is a complex concept that can be interpreted differently by users due to different times, places, and usage patterns; it has different meanings to consumers and even marketers. It has therefore been described as an abstract concept [33]. This concept has been applied in a wide range of fields, such as economics, social sciences, accounting, finance, strategy, product management, information systems, marketing, and tourism studies [34]. Gallarza and Saura [35] stated that value is the key to gaining competitive advantage and that the main purpose of marketing is to emphasize the importance of value. Lin et al. [2] stated that value is the criterion that guides individuals’ daily activities, directly influencing their measurement and evaluation of people, events, and objects, and that value is a fundamental determinant of consumer attitudes and behaviors. However, as each person has different innate and acquired conditions and life experiences, the formation of values will be affected differently. Therefore, individuals each have their own values and value systems. Under the same objective conditions, people with different values and value systems have different motivational patterns and produce different behaviors, which makes value an important predictor of behavior. Gutman [36] argued that interviews can be used to explore the deepest values and needs of consumers; higher and deeper terminal values can be obtained through a painstaking, step-by-step process from the explicit to the abstract and from the lowest external level to the highest internal level. Jeng et al. [6] argued that value is the process of achieving the goals and objectives desired by customers through the consumption process, as well as the assessments and cognitive preferences for product attributes and the performance and consequences of attributes. When exploring the decision-making process of older adults participating in virtual reality recreation activities, researchers found, through in-depth interviews, that the terminal values of older adults for participation in virtual leisure activities were good memories, a sense of social belonging, and improved quality of life.

### 2.4. Means–End Chains (MECs)

MECs explore the important meanings that individuals/consumers attribute to products/events through their cognitive schemas in order to describe their innermost thoughts about the products or events [37,38,39]. MECs are based on the relationship between the attributes of the product/event, associated consequences/feelings after consumption, and the personal values achieved [36]. Attributes (As) are characteristics of a good or service that are known and perceived by consumers [40], including both specific and abstract attributes, such as packaging, price, quality, brand, and the seller’s service and reputation. Consequences (Cs) refer to the direct or indirect effects on consumers after they buy or use a product and can be divided into functional consequences and psychological consequences. Functional consequences represent the direct benefits obtained after consumption, whereas psychological consequences represent the psychological or social consequences that result from use [41]. Personal values (Vs) are the psychological expressions of a consumer’s attempts to achieve important life goals and can be divided into instrumental values and terminal values. Instrumental values are ways of behaving to achieve a final goal. Terminal values are the perceived desirable end states after consumption [42]. Attributes are the means by which consequences satisfy personal values [6]. The main purpose of this theory is to explain the reasons for consumers’ decision-making choices and behaviors through A/C/V links.

To form the MEC structure, Reynolds and Gutman [43] employed the laddering interview technique originally developed by Hinkle [44] to understand how consumers translate product attributes into meaningful links with the self in accordance with MECs. This technique can be divided into soft laddering and hard laddering [45,46]. Soft laddering is conducted through one-on-one in-depth interviews, allowing respondents to answer questions without restrictions, using the free elicitation method to obtain data. It is not suitable for a large sample size, but a minimum of 20 samples are required [40]. Hard laddering can be done by phone, e-mail, or self-administered questionnaires, collecting information on the hierarchical order of attributes, consequences, and values through the answers from respondents on each level. This approach has a lower cost and is conducive to a large sample size of over 59 samples [45,47,48].

All interviews are content analyzed, coded as A, C, and V variables, and incorporated into an implication matrix. The matrix provides detailed information about the link frequency for each A–C and C–V. Finally, these A–C and C–V linking processes are shown in the hierarchical value map (HVM). To avoid over-complicating the HVM, a cut-off level is set. A high cut-off level produces a simple map involving less information and fewer connections that are easier to interpret. A low cut-off level produces a complex map containing a large amount of information that is more difficult to interpret [49]. Generally, the cut-off level is set depending on the sample size of the study. For a sample size of 20 samples, the cut-off level is a frequency of at least twice before it appears in the HVM [43]. The results of the MEC analysis have been used to develop effective strategies in the fields of tourism, leisure/recreation, education, and virtual leisure [2,38,39,50].

## 3. Research Methods

### 3.1. Data Collection

As this study is exploratory in nature, the soft laddering interviews were used as the main data collection method. The respondents were older adults over the age of 60, and the location was a senior citizens’ learning center in Taichung, Taiwan. Senior citizens’ learning centers provide seniors with the opportunity to continue learning and participating in activities that enhance quality of life and promote active aging. The researchers went to the aforementioned location to find older adults who were willing to participate in the study. A total of 40 older adults agreed to participate in the study, and data collection was conducted in two stages.

Stage 1: A smart bracelet was given to each of the 40 older adults willing to wear a Xiaomi bracelet. Participants were taught how to use the smart bracelet and were informed of the purpose of the study. Participants were taught about the function of a pedometer, heartbeat record, sleep quality monitoring, and sedentary reminder. The research assistants ensured that every participant knew how to use these function and how to record the information our research team required. All participants wore the bracelets for three months, from 8 October 2019 to 8 January 2020.

Stage 2: After three months, the interviewers contacted each of the 40 participants who had worn the smart bracelets to arrange a time and place to carry out a one-to-one in-depth interview. The purpose of the interview and how the interview would be conducted were explained to each of the participants, with two interviewers demonstrating the conduct of the interview. Each interview lasted between approximately 40 and 60 min. The interviews focused on the links between the attributes of the smart bracelet and the consequences and values obtained post-experience. First, we assessed whether the answers provided by the participants referred to the smart bracelet attributes by asking, “Why do you like to wear the Xiaomi bracelet?” Next, the in-depth interviews were conducted on the basis of each of these attributes. The questions were as follows: “Why is this attribute important to you?”, “What utilities or benefits can this attribute bring you?”, and “What values can you feel that these utilities or benefits bring to your own life?” Interviewers systematically guided respondents from attributes to consequences and then from consequences to personal values. The interview process ended when respondents were unable to continue answering questions.

### 3.2. Data Analysis

Content analysis was carried out on the data collected from the interviews. Content analysis is the objective, systematic, and quantitative description of the manifest content of communication [51]. This method is the primary method for analyzing communication content in communication studies. However, because the explicit and implicit content of interview data can be systematically organized and synthesized, and the data can be classified and coded, this method has been gradually adopted by other social science disciplines. The present study first classified the same phrases from the verbatim transcription. Following a discussion with three scholars who were familiar with MEC theory and had experience in researching smart health wear, we excluded the unsuitable phrases and categorized the data, assigning a name to each factor. The next step was coding—distinguishing the levels of MEC attributes, consequences, and values (A/C/V) according to inter-subjectivity [45] and determining the operational definitions of attributes, consequences, and values. Finally, we carried out reliability analysis, with the following test:

1. Intercoder agreement:

$$R=\frac{2M}{N_{1}+N_{2}}$$

*R* = Intercoder agreement

*M* = Frequency of matches between two coders

*N*_1_ = Coding frequency of first coder

*N*_2_ = Coding frequency of second coder

2. Compound reliability:

$$CR=\frac{N\;\times Average\;intercoder\;agreement}{1+\left[\left(N-1\right)\times Average\;intercoder\;agreement\right]}$$

*CR* = Composite reliability

*N* = Number of coders

## 4. Research Results

### 4.1. Description of the Interview Sample

The number of valid samples was 40 older adults, including 24 males (60%) and 16 females (40%). In terms of age, the 66–70-year-old group was the largest, with 24 respondents, accounting for 60% of respondents. In terms of occupation, retirees made up the largest group, with 18 respondents, accounting for 45% of the total. This was followed by people from the service industry, with 10 respondents, accounting for 25% of the total. In terms of level of education, senior high school and vocational school made up the largest group, with 32 respondents, accounting for 80% of the total. Finally, 24 respondents (60%) lived with family members, while 12 respondents lived with spouses. Detailed information is shown in Table 1.

### 4.2. Reliability of Interview Data and Intercoder Agreement

#### 4.2.1. Coding Results

From the verbatim transcripts of the 40 participants in this study, following a discussion among the three coders (a, b, c), unsuitable phrases were excluded. The remaining phrases were then categorized, producing a total of 15 factors. Each factor was named. The categories were real-time information feedback, safe use, comfortable wear, clear screen, correct data, cheap price, learning about smart products, understanding technology applications, increased health awareness, relaxation, satisfying curiosity, healthier bodies, improved quality of life, a sense of social belonging, and better relationships with others, as shown in Table 2.

#### 4.2.2. Reliability and Intercoder Agreement

According to the definitions of factors in Table 2, the contents of the interviews were defined in terms of attributes, consequences, and values. Keywords were categorized, and coding analysis was carried out after obtaining word stems and classification rules. The 15 factors were then named according to their characteristics, coded, and further classified into the three levels of attributes, consequences, and values.

There were six attributes: A1 real-time information feedback, A2 safe use, A3 comfortable wear, A4 clear screen, A5 correct data, and A6 cheap price.

There were five consequences: C1 learning about smart products, C2 understanding technology applications, C3 increased health awareness, C4 relaxation, and C5 satisfying curiosity.

There were four values: V1 healthier bodies, V2 improved quality of life, V3 a sense of social belonging, and V4 better relationships with others. Finally, three experts were asked to assess each other’s reliability, as shown in Table 3.

Using the Kappa index to calculate the inter-judge agreement between pairs of codes as a measurement index [52], Kassarjian [53] believed that a reliability greater than 0.85 was satisfactory. In the present study, for the coding of attributes, consequences, and values in Table 3, the intercoder agreement was 0.89, 0.96, and 0.85, respectively, and the overall reliability was 0.96, which met the level suggested by experts, demonstrating that the reliability of the data collected in the present study was quite high, as shown in Table 4.

#### 4.2.3. Definitions of Attributes, Consequences, and Values

Three specific attributes were extracted from the content analysis: real-time information feedback, safe use, and correct data; the three abstract attributes were comfortable wear, clear screen, and cheap price. Of the five consequences, three were functional consequences: learning about smart products, understanding technology products, and increased health awareness; two were psychological consequences: relaxation and satisfying curiosity. Of the four values, two were instrumental values: improved quality of life and a sense of social belonging; two were terminal values: healthier bodies and better relationships with others.

In terms of the frequency of responses, of the six attributes, comfortable wear was mentioned the most often (12 times), followed by real-time information feedback and correct data (8 times each). Of the five consequences, relaxation was mentioned the most often (24 times), followed by understanding technology products (14 times), and increased health awareness and satisfying curiosity (10 times each). Of the four value descriptions, healthier bodies was mentioned the most often (20 time), followed by a sense of social belonging (16 times) and improved quality of life (14 times). Higher frequencies indicated the factors that were more important to consumers, as shown in Table 5.

#### 4.2.4. Implication Matrix

The implication matrix, an important tool for integrating the link frequencies, was used to produce the HVM. According to the chain relationships between attributes, consequences, and values produced by laddering, the implication matrix was constructed. Interviews with the respondents can generate an “attribute–consequence–value” relationship ladder. The numbers in the matrix indicate the frequency of the direct and indirect chain links between attributes, consequences, and values as shown in the matrix columns (Reynolds et al., 2001). The number before the semicolon (;) represents the frequency of the direct chain links between different factors, while the figures after the symbol indicate the frequency of the indirect chain links between the factors. The figures indicate the strength of the chain link between the factors.

As shown in Table 6, the 40 respondents constructed a total of 120 ladders, with each respondent mentioning 6 ladders on average. In the links between attributes (A) and consequences (C), the link between “A3. Comfortable wear” and “C3. Increased health awareness” and the link between “A5. Correct data” and “C5. Satisfying curiosity” were the strongest (8;0), reaching a moderate level. In the links between consequences (C) and values (V), there was a strong link between “C4. Relaxation” and “V2. Improved quality of life” (12;2) and a moderate link between “C4. Relaxation” and “V3. A sense of social belonging” (10;0). At the consequences (C) level, there was a strong link between the functional consequence of “C2. Understanding technology products” and the psychological consequence of “C4. Relaxation” (14;0). In addition, there was a moderate link between “C3. Increased health awareness” and “C4. Relaxation” (6;0).

#### 4.2.5. Creating the Hierarchical Value Map

**Figure 1:**
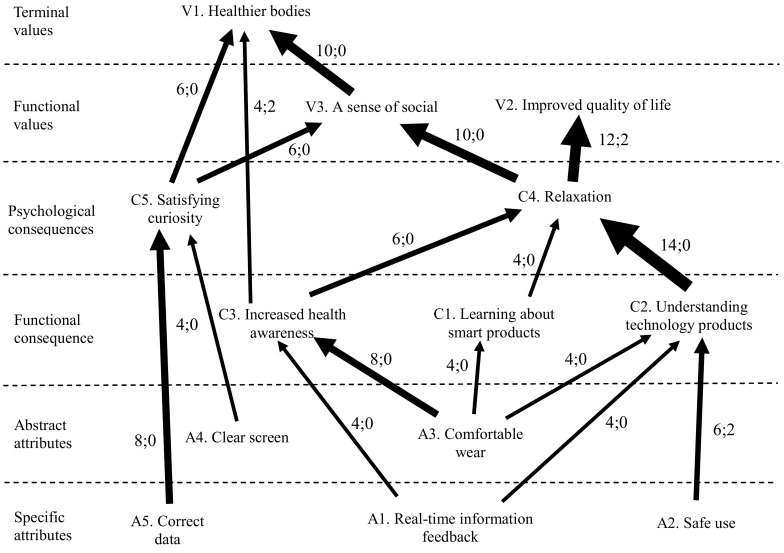
Hierarchical value map for mobile health devices.

To ensure a clear display of the important A/C/V chain relationships, only A/C/V associations with a frequency of three or more links are shown in the HVM (see Figure 1). The frequency of the links between factors is indicated with arrows of differing thickness, with thicker lines indicating stronger associations. Typically, an association with four or fewer links is considered weak, an association with between five and nine links is considered moderate, and an association of ten or more links is considered strong [54].

In the paths of linkages in Table 1, there are five main paths in the HVM for mobile health devices.

Path 1: From “safe use,” “real-time information feedback,” and “comfortable wear” to the consequences of “understanding technology products” and “relaxation,” obtaining values of “a sense of social belonging” and “healthier bodies.”

Path 2: From “correct data” and “clear screen” to the consequence of “satisfying curiosity,” obtaining the value of “healthier bodies.”

Path 3: From “comfortable wear” to the consequences of “learning about smart products” and “relaxation,” obtaining the value of “improved quality of life.”

Path 4: From “comfortable wear” and “real-time information feedback” to the consequence of “health awareness,” obtaining the value of “healthier bodies.”

Path 5: From “clear screen” to the consequence of “satisfying curiosity,” obtaining the value of “social belonging.”

### 4.3. Discussion

The present study used means–end chains to explore the value of using mobile health devices to older adults. What consequences do the specific and abstract attributes of smart bracelets produce for older adults? What values do these consequences have for older adults? Based on the HVM for mobile health devices in Figure 1, the present study offers the following three main findings.

Finding 1: In terms of attribute functions, older adults believe that smart bracelets as mobile health devices have the attributes of safe use, real-time information feedback, correct data, comfortable wear, and clear screen. Older adults have shown a high degree of cognitive, psychological, physiological, and sensory (e.g., vision, hearing, touch, and sensitivity) differences from young people when using smart bracelets [55]. Therefore, older adults believe that comfortable wear and clear screen are essential. Although the use of mobile health devices offers new solutions for the self-assessment of physiological performance and behavior, to date, most smart bracelets have not been developed to meet the needs of seniors. In terms of safety, a study by Hawley et al. [56] on the perceptions of health devices aimed at fall prevention found that the needs for control, independence, and safety are important motivations for using these devices. A study by Lin et al. [2] also identified safe use as an important activity attribute. In addition, activities are an important component of healthy aging. Activities should be incorporated into active exercise as a part of daily life, while smart bracelets should also be used to monitor individual behavior, such as giving sedentary reminders, indicating the calories burned, and checking for risks of functional decline. The collection of data on overall balance and physical activity in daily life, which also allows real-time feedback of data for analysis and interpretation, increases the motivation of older adults to change their health behaviors [5]. Real-time information feedback can increase physical activity, reduce sedentary time, and support the promotion of healthy lifestyles and more active activities [57]. Therefore, older adults consider that the real-time information feedback and correct data provided by smart bracelets as mobile health devices are important attributes.

Finding 2: In terms of outcome benefits, older adults that use smart bracelets as mobile health devices can achieve the benefits of increased health awareness, learning about smart products, understanding technology products, satisfying curiosity, and relaxation. A growing number of older adults are using smartphones and mobile health devices. Older adults who use technology and online services in their daily lives may also encounter a variety of access barriers, including a lack of familiarity and trust in new things and a lack of confidence in their own skills. In addition to physical changes, the aging process involves psychological and social changes. Factors such as sex, age, socioeconomic class, and the individual’s situation influence the demand for or the use of mobile health devices [58]. Therefore, if older adults are to accept mobile health devices, such devices must have obvious benefits for them and be consistent with their goals, expectations, and lifestyle [59]. Using mobile health devices can satisfy the curiosity of older adults. Understanding technology can help increase knowledge and enhance one’s own health awareness [14]. In other words, these outcome benefits have an impact on health literacy and health management.

Finding 3: In terms of value goals, older adults want to achieve the value goals of a sense of social belonging, improved quality of life, and healthier bodies. Research by Routasalo et al. [60] pointed out that social isolation, loneliness, and a diminished sense of well-being lead to a deterioration in health, producing an increase in medical services, medical costs, and mortality rates. Previous studies have also shown that the use of information and communication technology by older adults can prevent loneliness, social isolation, declining health, and frailty [61]. In recent years, mobile health devices with goal-setting and self-monitoring functions have become an important platform for providing health interventions. In particular, they can increase the duration and frequency of exercise for sedentary older adults [62]. Their content includes health-related knowledge, reminders, feedback and support, answering questions, and uploading self-monitoring data, including physiological aspects, such as blood pressure and blood sugar, and psychological indicators, such as self-efficacy. These devices provide older adults with an effective means of accessing health information and changing behavioral intentions, ultimately leading to changes in actual health behaviors and helping to improve the quality of life of older adults following retirement [7]. This shows that health is the one thing that older adults care about the most. However, previous studies have tended to focus on the use of devices for physical examinations, with few studies focusing on the firsthand experiences of older adults. The present study showed that having older adults wear and experience mobile health devices firsthand can provide them with the terminal value of long-term health promotion.

## 5. Conclusions

The arrival of smart bracelets as mobile health devices on the market reflects the contemporary importance of health consciousness. Further, older adults are an important market opportunity for these products. Currently, the design of wearable devices on the market for monitoring physiological values and recording is still mainly based on smart sports watches. Aside from the demand for these sports watches and their functions among the participants in outdoor sports, such as joggers and cyclists, most older adults do not have an in-depth knowledge of these products. Smart bracelets are becoming increasingly popular among older adults because of their simplicity and affordability. In the present study, it was found that in addition to safety, older adults also value comfort when wearing the bracelets and a clear screen. Older adults have difficulty viewing small screens due to deterioration in their eyesight. However, an obvious limitation of the market is that the technology has not been developed based on the needs of older users, so there are still no smart bracelets designed specifically for older users. Another important factor is the constant emergence and development of new technologies and the rapid replacement of existing products by new ones, which makes adoption more difficult. To move from treatment to prevention in response to an aging population, mobile health devices need to be developed based on the needs and preferences of older adults, allowing older adults to continue using such products and indirectly allowing them to remain independent and active for longer.

In addition, the accuracy of the data is also an important issue in the development of new products. Valid and reliable data must be provided for real-time feedback of information, risk detection, and prognostic results. Using smartphone apps and workout platforms for the systematic evaluation of diet improvement, physical activity, and preventive measures for sedentary behavior has been shown to be effective [31]. However, there is little evidence on the effectiveness of health monitoring using smart bracelets for functions such as health promotion and latent interest. While new algorithms for monitoring data are constantly being developed, the reliability and validity of these algorithms need to be carefully evaluated. Moreover, mobile health devices must monitor behavior over time and evaluate changes in individual behavior pre- and post-use based on preventive measures or changes in health status. In addition to providing insight into the effectiveness of preventive measures, these new data can facilitate the development of more effective preventive measures and provide a better direction for individualized prevention.

Finally, how is product “value” created? Due to their sound mental and physical functions, retired older adults are still closely connected to society and have certain social needs; therefore, they place more emphasis on the social, sharing, and entertainment functions of electronic products and mobile health devices. These older adults are concerned not only with physiological values, but also with their mental state and quality of life. Maintaining a happy mood, recording everyday life occurrences, sharing big and small things in life with friends or peers, and promoting healthier bodies are reasons why this group of healthy older adults would be willing to use mobile health devices. Instead of spending time trying to figure out what new features to add to a product, it would be preferable to look at aspects of the “user experience” that might bring more value.

For older adults, motivation and participation are the key factors in changing behavior. Using mobile health device services has the potential to promote change in health behavior over time. However, to date, little is known about effective behavioral change following use of of health promotion following long-term use. Therefore, follow-up studies can design monitoring measures to adapt to the needs and motivations of users according to the duration of use. In addition, the collection, computation, and storage of health behavior data provide opportunities for using big data. However, with the high possibility of using and sharing sensitive data, such as health conditions, locations, emotions, and social interactions, there are likely to be concerns about users’ privacy, security, and confidentiality. In addition, older people in different countries or territories have different attitudes around aging. Therefore, attributes, consequences, and values of smart bracelets in a hierarchical value map might different. Therefore, the values should be explored in different cultures. Even more, future studies can compare their results with those in this study to find similarity and differences. Finally, the present study used qualitative research methods to explore the value of mobile health devices for older adults through laddering interviews, which enabled us to gather generalized data from a small number of respondents. It is suggested that future research can use soft laddering to conduct in-depth interviews and obtain data and then use the data to create quantitative questionnaires to carry out large-scale surveys; this would enable the collection of data from a greater number of samples as a reference for relevant units.

## Figures and Tables

**Table 1:** Description of the interview sample.

Type	Item	Number of People	Percentage
Sex	Male	24	60%
	Female	16	40%
Age	60–65	6	15%
	66–70	24	60%
	71–75	6	15%
	Over 76	4	10%
Level of education	Elementary school	2	5%
	Junior high school	6	15%
	Senior high school and vocational	16	40%
	Junior college or above	16	40%
Occupation	Military and government personnel	8	20%
	Service industry	4	10%
	Manufacturing industry	10	25%
	Retirees	18	45%
Monthly disposable income	NT$20,000 or less	6	15%
	NT$20,001–NT$40,000	14	35%
	NT$40,001–NT$60,000	14	35%
	More than NT$60,001	6	15%
Housing situation	Living with spouse	12	30%
	Living with family members	24	60%
	Living alone	2	5%
	Other	2	5%

**Table 2:** Names and definitions of factors.

Name of Factor	Definition of Factor	Content
1. Real-time information feedback	Real-time display of data	Steps, sleep quality, heartbeat, heart rate, calories burned
2. Safe use	Device is not harmful to the body	Radiation, battery, usage method
3. Comfortable wear	Device does not cause feelings of constraint when worn	Made from soft materials, small size
4. Clear screen	The bracelet screen is clear	Color, symbols, font size
5. Correct data	Monitoring data accuracy	Heartbeat, calories burned, sleep quality
6. Cheap price	Long-term use represents excellent value	Low failure rate, many functions
7. Learning about smart products	Understanding the significance of smart products in health education	Experience of different functions
8. Understanding technology applications	Understanding the impact of technology on health	Health, food, travel, housing, and recreation
9. Increased health awareness	Understanding physical conditions and how to prevent decline	Exercise motivation, health monitoring
10. Relaxation	Eliminating fatigue, causing a relaxed feeling	Physical and mental relaxation
11. Satisfying curiosity	Being driven to explore new technologies	Curiosity, stimulation of creativity
12. Healthier bodies	Having balance in mind and body, living longer	In good spirits, in good health
13. Improved quality of life	Living comfortably, having fun, achieving goals	Happy, joyful, satisfied
14. A sense of social belonging	Fitting into society, being respected	Harmonious society, caring society
15. Better relationships with others	Having a better understanding of other people, living in harmony with other people	Interaction, communication

**Table 3:** Coding of factors.

Factors		Attributes			Consequences			Values		
		a	b	c	a	b	c	a	b	c
Attributes	A1. Real-time information feedback	O	O	O						
	A2. Safe use	O	O	O						
	A3. Comfortable wear	×	O	O						
	A4. Clear screen	O	O	O						
	A5. Correct data	O	O	×						
	A6. Cheap price	O	O	O						
Consequences	C1. Learning about smart products				O	O	O			
	C2. Understanding technology applications				O	O	O			
	C3. Increased health awareness				×	O	O			
	C4. Relaxation				O	O	O			
	C5. Satisfying curiosity				O	O	O			
Values	V1. Healthier bodies							O	O	O
	V2. Improved quality of life							×	O	O
	V3. A sense of social belonging							O	O	O
	V4. Better relationships with others							O	O	O

O: Indicates agree; ×: Indicates disagree.

**Table 4:** Intercoder agreement and reliability.

Coder	a:b	b:c	c:a
Intercoder agreement	0.89	0.96	0.85
Average level of agreement	0.90		
Overall reliability	0.96		

**Table 5:** Definitions of attributes, consequences, and values.

Attributes (Number of Times Mentioned)		Consequences (Number of Times Mentioned)		Values (Number of Times Mentioned)	
Specific attributes	A1. Real-time information feedback (8) A2. Safe use (6) A5. Correct data (8)	Functional consequences	C1. Learning about smart products (4) C2. Understanding technology products (14) C3. Increased health awareness (10)	Functional values	V2. Improved quality of life (14) V3. A sense of social belonging (16)
Abstract attributes	A3. Comfortable wear (12) A4. Clear screen (4) A6. Cheap price (2)	Psychological consequences	C4. Relaxation (24) C5. Satisfying curiosity (10)	Terminal values	V1. Healthier bodies (20) V4. Better relationships with others (2)

**Table 6:** Implication Matrix (*n* = 40).

Factor	C1	C2	C3	C4	C5	V1	V2	V3	V4	Total
A1	0;0	4;0	4;0	0;6	0;0	0;6	0;0	0;4	0;2	8;18
A2	0;0	6;2	0;0	0;6	0;0	0;4	0;4	0;4	0;0	6;20
A3	4;0	4;0	8;0	0;16	0;0	0;4	0;12	0;2	0;2	16;36
A4	0;0	0;0	0;0	0;0	4;0	0;2	0;0	0;2	0;0	4;4
A5	0;0	0;0	0;0	0;0	8;0	0;4	0;0	0;4	0;4	8;12
A6	2;0	0;2	0;0	0;0	2;0	0;0	0;0	0;2	0;2	4;6
C1	0;0	0;0	0;0	4;0	0;0	0;0	0;4	0;0	0;4	4;8
C2	0;0	0;0	0;0	14;0	0;0	0;6	0;8	0;8	0;0	14;22
C3	0;0	0;0	0;0	6;0	0;0	4;2	0;4	0;2	0;2	10;10
C4	0;0	0;2	0;0	0;0	0;2	0;10	12;2	10;0	2;0	24;16
C5	0;0	0;0	0;0	0;0	0;0	6;0	0;0	6;0	0;2	12;2
V1	0;0	0;0	0;0	0;0	0;0	0;0	0;2	0;0	0;2	0;4
V2	0;0	0;0	0;0	0;0	0;0	0;0	0;0	0;0	0;0	0;0
V3	0;0	0;0	0;0	0;0	0;0	10;0	0;0	0;0	0;0	10;0
V4	0;0	0;0	0;0	0;0	0;0	0;0	0;0	0;0	0;0	0;0
Total	6;0	14;6	12;0	24;28	14;2	20;38	12;36	16;28	2;20	120;158
